# Developing Hollow-Channel Gold Nanoflowers as Trimodal Intracellular Nanoprobes

**DOI:** 10.3390/ijms19082327

**Published:** 2018-08-08

**Authors:** Sunjie Ye, May C. Wheeler, James R. McLaughlan, Abiral Tamang, Christine P. Diggle, Oscar Cespedes, Alex F. Markham, P. Louise Coletta, Stephen D. Evans

**Affiliations:** 1 School of Physics and Astronomy, University of Leeds, Leeds LS2 9JT, UK; s.ye@leeds.ac.uk (S.Y.); may.wheeler1@gmail.com (M.C.W.); phat@leeds.ac.uk (A.T.); o.cespedes@leeds.ac.uk (O.C.); 2 Leeds Institute for Biomedical and Clinical Sciences, University of Leeds, Leeds LS9 7TF, UK; c.p.diggle@leeds.ac.uk (C.P.D.); a.f.markham@leeds.ac.uk (A.F.M.); p.l.coletta@leeds.ac.uk (P.L.C.); 3 School of Electronic and Electrical Engineering, University of Leeds, Leeds LS2 9JT, UK; J.R.McLaughlan@leeds.ac.uk; 4 Leeds Institute of Cancer and Pathology, University of Leeds, Leeds LS9 7TF, UK

**Keywords:** gold nanoflowers, intracellular nanoprobes, surface plasmon resonance, SERS, fluorescence, dark-field

## Abstract

Gold nanoparticles-enabled intracellular surface-enhanced Raman spectroscopy (SERS) provides a sensitive and promising technique for single cell analysis. Compared with spherical gold nanoparticles, gold nanoflowers, i.e., flower-shaped gold nanostructures, can produce a stronger SERS signal. Current exploration of gold nanoflowers for intracellular SERS has been considerably limited by the difficulties in preparation, as well as background signal and cytotoxicity arising from the surfactant capping layer. Recently, we have developed a facile and surfactant-free method for fabricating hollow-channel gold nanoflowers (HAuNFs) with great single-particle SERS activity. In this paper, we investigate the cellular uptake and cytotoxicity of our HAuNFs using a RAW 264.7 macrophage cell line, and have observed effective cellular internalization and low cytotoxicity. We have further engineered our HAuNFs into SERS-active tags, and demonstrated the functionality of the obtained tags as trimodal nanoprobes for dark-field and fluorescence microscopy imaging, together with intracellular SERS.

## 1. Introduction

Raman spectroscopy represents a highly informative technique that enables optical detection and identification of vibrational modes corresponding to individual bonds. The weak signal from conventional Raman scattering makes it difficult to achieve excellent sensitivity, which is indeed desired for spectral measurements of biological systems, for example, single cells. Surface-enhanced Raman spectroscopy (SERS) combines the abundant chemical fingerprint information of Raman spectroscopy, and a greatly improved sensitivity by plasmon-enhanced excitation and scattering [1,2]. In particular, Raman spectra of molecules adsorbed on the surfaces of noble metal (gold or silver) nanoparticles can be enhanced by up to 14 orders of magnitude [3]. Gold nanostructure-based intracellular SERS provides a powerful and promising tool, enabling ultrasensitive and structurally-selective detection of native chemicals, such as DNA, proteins, lipids etc., and the monitoring of their intracellular distributions [4,5,6,7]. Gold “nanoflowers” are a type of engineered nanostructure, with multiple branches that give the overall shape of a flower [8,9,10,11]. Owing to the abundant sharp tips serving as “hot spots” for localized near-field enhancements, together with their large surface area-to-volume ratios, Au nanoflowers exhibit larger SERS activity than nanoparticles with smooth surfaces [12,13]. Furthermore, the spiky features of nanoflowers can promote penetration of the cell membrane and enhance cell internalization [14]. In addition, the surface plasmon resonance of Au nanoflowers can be readily tuned into the near-infrared region of the tissue-transparent window (650–900 nm) [15], which uses a lower-energy laser source, and thus facilitates non-invasive intracellular SERS, with great potential for in vivo SERS imaging [16]. It has also reported that multiple-branched Au nanoflowers are effective in photothermal therapy [17], and the therapeutic outcome of photothermal therapy can be monitored in real-time by gold nanoparticles-based intracellular SERS [18]. Hence, Au nanoflowers-based nanotags hold potential towards theranostic nanosystems, which integrate imaging functions and photothermal therapy to realize imaging-guided phototherapy. Despite several advances in the development of Au nanoflowers as intracellular SERS nanoprobes, the majority of the synthesis methods involve surface capping agents (for example, widely-used surfactant cetyltrimethylammonium bromide, CTAB) for inducing the anisotropic nanocrystal growth to form the branches [19]. On the one hand, the capping agents on the surface produce the background signal arising from their own chemical fingerprint and hinder the interaction of the analyte with the Au surface, thus, resulting in a loss of sensitivity [20]. On the other hand, free CTAB in the dispersion is known to have high cytotoxicity [21], limiting the intracellular functions associated with gold nanoflowers. To address these issues, we have recently developed a one-step surfactant-free approach to fabricating novel nanoflowers with an open hollow channel [22]. In our method, the formation of spiky features relies on the anisotropic nanocrystal growth induced by an auto-degradable nanofiber template, which also leads to the generation of an open hollow channel. More specifically, during the formation of our hollow-channel gold nanoflowers (HAuNFs), the template of methyl orange (MO)-FeCl_3_ nanofiber induces the anisotropic growth of the primary Au nanocrystals nucleated on the template, resulting in the production of the spikes/branches of the flower-shape. The as-formed Au nanoflowers (NFs) then acted as a catalyst for degrading MO in the presence of FeCl_3_, excess ascorbic acid (AA, reducing agent), and oxygen in the solution, causing the disassembly of the MO-FeCl_3_ nanofiber template, leaving an open hollow channel in the center of the Au NF, and no residue of MO on the surface of the obtained HAuNFs or in the dispersion. As a result, our hollow-channel gold nanoflowers (HAuNFs) have several potential advantages over conventional nanoflowers for the application as intracellular SERS nanoprobes, including: (1) The existence of the hollow channel; in addition to increasing the total surface area, the open cavity renders the interior space accessible. The facing inner walls have a distance of ca. 10 nm, determined by the thickness of the nanofiber template, hence, forming nanogaps, which can give rise to the substantial enhancement of the plasmon electromagnetic field [23,24] and contribute to the large single-particle SERS activity; (2) the relatively “clean surface” owing to the surfactant-free synthesis obviates the background signal from the surface capping layer and makes the Au surface more easily accessible for the analytes; and (3) better biocompatibility due to the preparation protocols without using CTAB. In addition, the surfactant-free synthesis may also enable easy surface modification [25], which endows Au nanoflowers with other imaging capabilities.

In this study, we have fabricated HAuNFs, with the maximum extinction around 640 nm, to match the excitation wavelength in Raman measurements. In vitro tests on RAW 264.7 macrophage cell lines suggest that the as-prepared HAuNFs exhibit efficient cellular uptake and good bio-compatibility. We have further modified these HAuNFs with fluorescent dyes, which also plays the role of Raman reporter molecules. The resultant SERS nano-tags have demonstrated potency as trimodal nanoprobes, integrating the dark-field imaging capability due to the inherent scattering properties of HAuNFs, and the fluorescence imaging function originating from the dye molecules, together with the intracellular SERS activity owing to the interaction of HAuNFs and Raman reporter molecules.

## 2. Result and Discussion

### 2.1. Fabrication and Characterization of HAuNFs with Desired SPR Propertes

**Figure 1:**
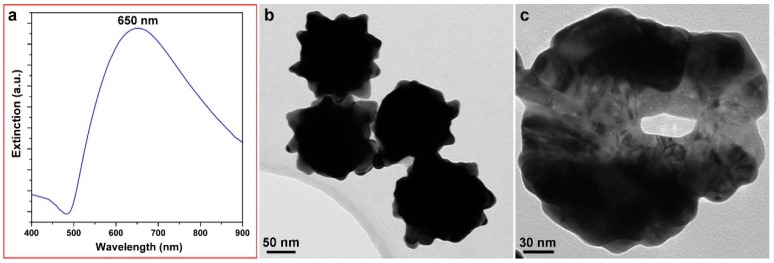
(**a**) UV-vis spectrum of hollow-channel gold nanoflowers (HAuNFs) synthesized with ascorbic acid (AA). (**b**) TEM image showing multiple branches on the outer surface; (**c**) TEM image in an orientation showing the hollow channel in the center.

The SERS effect of Au nanostructures is closely related with their surface plasmon resonance (SPR), and better matching between the SPR absorbance peak and the Raman excitation wavelength leads to greater enhancement [26]. Our previous work has shown that the SPR of HAuNFs can be modulated by tailoring the topology/roughness via changes of the concentration of the reducing agent, ascorbic acid (AA), in the synthesis [22]. In the current work, given the peak shift after subsequent surface modification (which will be discussed in Section 2.3), we have carefully optimized the concentration of AA so that the final HAuNFs-based SERS tag has a wavelength nearly the same as the laser excitation wavelength of 633 nm. As shown in Figure 1a, the synthesis with 125 mM AA yielded HAuNFs with an SPR of around 650 nm. The obtained HAuNFs have an overall size of (189 ± 12) nm and exhibit a flower-shape, with a quasi-spherical core surrounded by a number of spikes (Figure 1b). The inner cavity can be clearly observed (Figure 1c) when the nanoflower is lying on the TEM (Transmission Electron Microscope) grid, with the hollow channel aligning along the direction of the electron beam.

### 2.2. Assessment of Cellular Uptake and Cytotoxicity of HAuNFs

**Figure 2:**
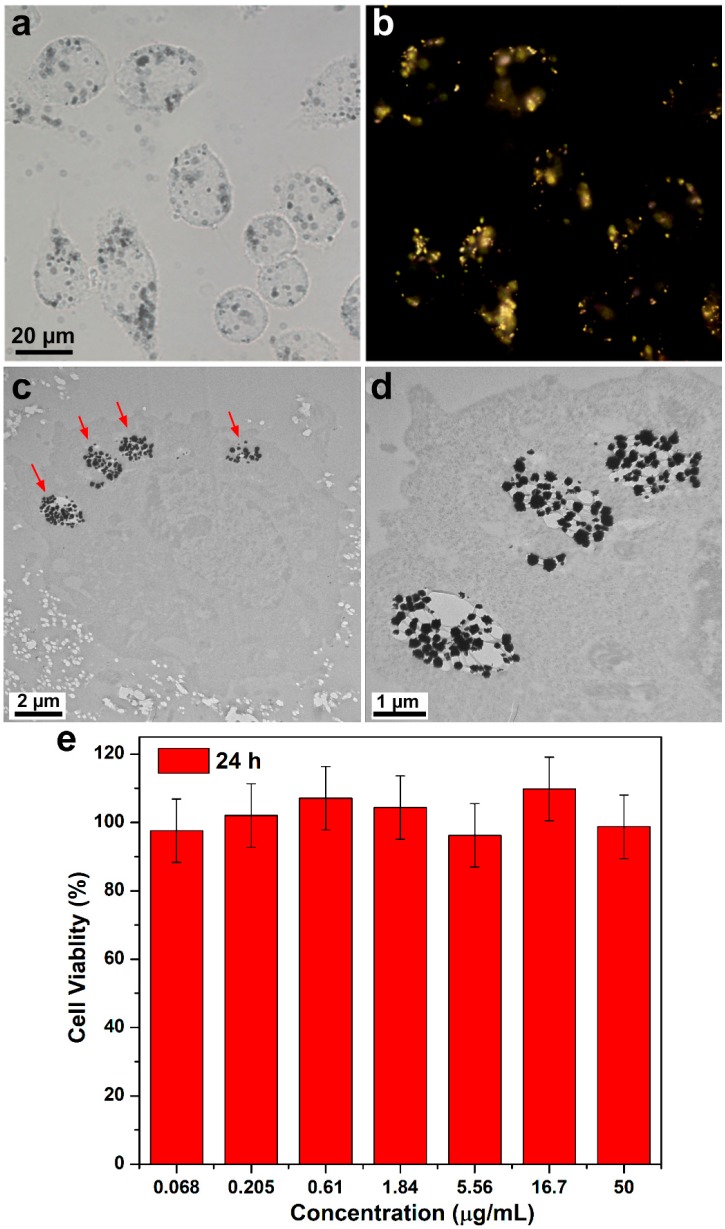
(**a**) Representative bright-field microscopy and (**b**) corresponding dark-field microscopy image of RAW macrophage cells treated with HAuNFs. Sectioned TEM images of a fixed Raw 264.7 cell treated with HAuNFs: (**c**) Low and high (**d**) magnification. The red arrows in (**c**) indicate HAuNFs; (**e**) cell viability of Raw 264.7 cells treated with varying concentrations of HAuNFs. Results are shown as mean ±SD (*n* = 6) as determined using Cell Counting Kit-8 (CCK-8) assays.

We have chosen Raw 264.7 macrophage cells to perform in vitro assessment of HAuNFs, Macrophages have emerged as key cellular targets for novel therapeutic methods and molecular imaging [27]. Figure 2b show optical microscopy images of RAW 264.7 cells after being treated with the medium containing HAuNFs for 24 h, fixed with paraformaldehyde/DPBS (Dulbecco’s Phosphate Buffered Saline) and washed with DPBS several times. The bright-field microscopy image (Figure 2a) reveals that the cells treated with HAuNFs are well attached on the cover slips and maintain their normal morphology, indicating a good biocompatibility of the HAuNFs. The dark field microscopy image (Figure 2b) exhibits the colocalization of the cells and the scattering signals form the HAuNFs. Notably, HAuNFs are found to be distributed in the cytoplasm with the absence of a signal at the nuclear regions instead of being randomly associated with the cells, which may suggest they have been internalized as opposed to being adhered on the cell surface [28]. This observation was further verified by the TEM images of the sectioned cells treated with HAuNFs. Figure 2c,d show that the HAuNFs have entered the cell and were mainly located in the vacuoles, revealing the internalization undergoes the pathway of endocytosis or specialized phagocytosis of macrophage cells. We tested the cytotoxicity of HAuNFs using Raw 264.7 cells with different doses of HAuNFs. After 24 h of incubation with HAuNFs, the cells possess a viability over 90% at all the tested dosages, which shows our HAuNFs have negligible cytotoxic effects on Raw 264.7 cells. These results, coupled with the large single-particle SERS activity of our HAuNFs observed in our previous study [22], have demonstrated the potential of them to be explored as safe and effective intracellular SERS nanoprobes. Moreover, the surfactant-free synthesis approach has endowed our HAuNFs with a “clean surface”, enabling us to perform easy surface modification for constructing HAuNFs-based SERS nanotags.

### 2.3. Surface Modifications of HAuNFs to form SERS Nanotags

**Figure 3:**
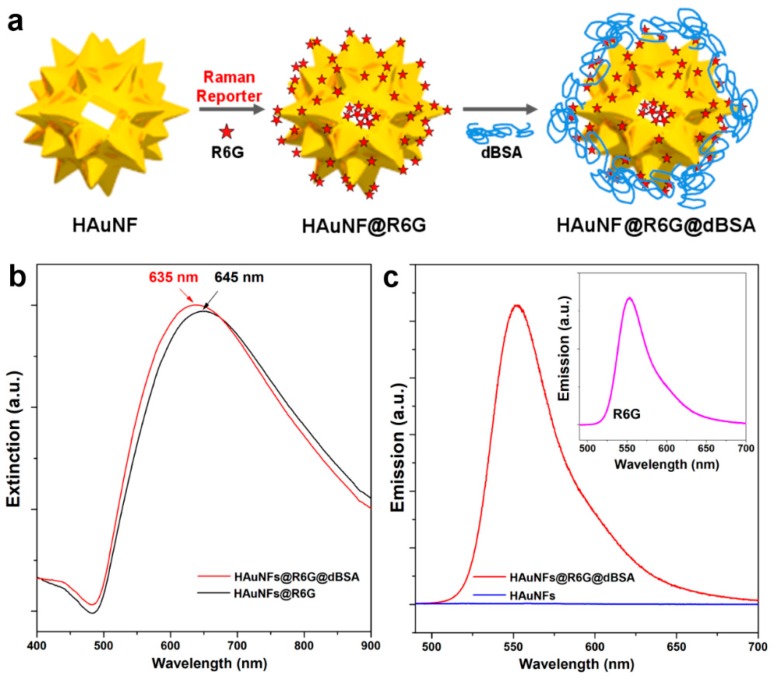
(**a**) Schematic representation of the surface modification of HAuNFs to form surface-enhanced Raman spectroscopy (SERS) nanotags (HAuNFs@R6G@dBSA); (**b**) UV-vis spectra of HAuNFs@R6G and HAuNFs@R6G@dBSA; (**c**) emission spectra (Excitation wavelength: 470 nm) of HAuNFs, HAuNFs@R6G@dBSA, and the aqueous solution of R6G (Inset of **c**).

Figure 3a schematically illustrates the surface modifications of HAuNFs to form SERS nanotags. The as-prepared HAuNFs were first encoded with the Raman reporter, Rhodamine 6G (R6G), and then coated with a layer of denatured bovine serum albumin (dBSA). In this nanotag design, R6G dye molecules are absorbed on the Au surface via electrostatic interactions as well as delocalized π-electrons, playing dual roles as the Raman reporter and fluorescent probes [7]. DBSA has been previously reported as providing Au nanoflower-dye nanoconjugates with good stability, owing to Au-S bonding formed between the Au surface and -SH groups in 35 Cys residues, together with the good steric protection imparted by the bulkiness of the protein [12]. As seen in Figure 3b, the SPR of HAuNFs shifted from 650 nm to 645 nm following the treatment with R6G, and then to 635 nm after being treated by dBSA, indicating successful conjugation formed in each step of the surface modification. The observed blue-shift after conjugation with R6G and dBSA can be ascribed to the combined effect of the changes in the refractive index and the surface charges. The plasmon resonances of the star/flower shaped gold nanostructures result from the hybridization of plasmons associated with the core and the individual nano-tips [29], and the nano-tips can be considered as nanorods. Gold nanorods possess a weak transverse band located at ca. 520 nm, and a strong longitudinal band [30]. The introduction of a dye and polymer layer leads to alternations of the local refractive index and surface charges, which can cause the blue-shift of the longitudinal band [31], hence, contributing to the blue shift of the plasmon peak of HAuNFs. Importantly, the SPR position (635 nm) of the resultant HAuNFs@R6G@dBSA nanotags ideally suits the 633 nm laser excitation of the Raman measurement. When measured under equivalent conditions (e.g., Au concentration, excitation wavelength, scan slit, etc.), the HAuNFs showed no fluorescent peak, whereas HAuNFs@R6G@dBSA exhibited a strong peak at 551 nm in the fluorescent spectrum (Figure 3c) in accordance with the fluorescent properties of R6G (Inset of Figure 3c). These favorable spectral features of HAuNFs@R6G@dBSA, including a strong fluorescence and desired SPR for SERS, have motivated us to evaluate the intracellular imaging functions of HAuNFs@R6G@dBSA.

### 2.4. Investigation of Bright-Field, Dark-Field, and Fluorescent Imaging Capability of HAuNFs-Containing Nanoconjugates

**Figure 4:**
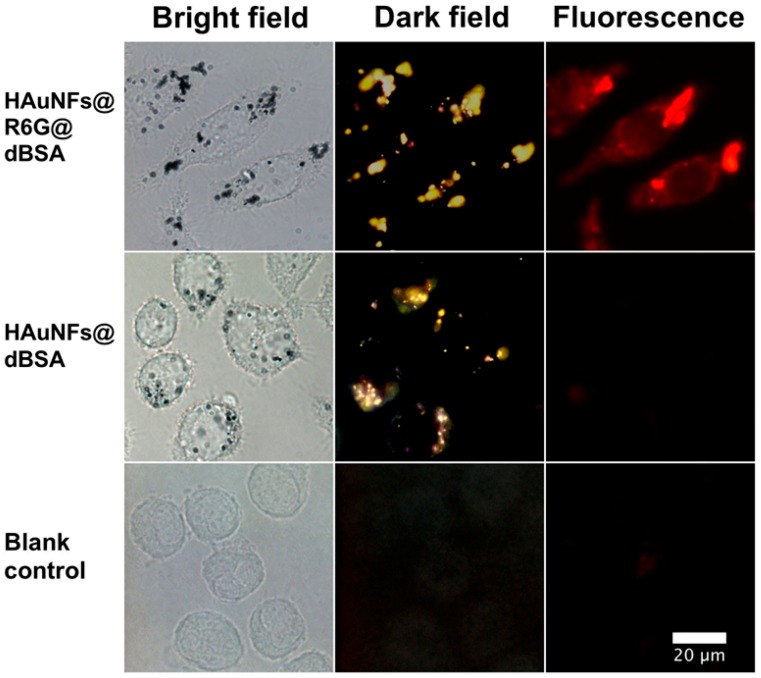
Bright-field, dark-field, and fluorescent microscopy images of RAW 264.7 cells after 12 h of incubation in a medium containing HAuNFs@R6G@dBSA, HAuNFs@dBSA, or medium only as a blank control. (Wider-view bright-field microscopy images of cells treated with HAuNFs@R6G@dBSA or HAuNFs@dBSA are shown in Supplementary Materials, Figures S1 and S2.)

Figure 4 shows the microscopy images of RAW 264.7 cells after different treatments. The cells incubated with medium can only be resolved in the bright field microscopy image, and showed no signal in the dark-field or fluorescence microscopy images. In contrast, the cells treated with HAuNFs-containing nanoconjugates showed black dots in the bright field microscopy images, with the contrast originating from the presence of nanoconjugates. At the same time, the dark-field images of these cells show strong yellow scattering signals from the HAuNFs. In particular, the cells incubated with HAuNFs@R6G@dBSA also demonstrated remarkable signals in the fluorescence images. The good spatial agreement of the signals observed in the dark-field and fluorescence microscopy and the absence of a dispersed fluorescence signal suggest the nanotags (HAuNFs@R6G@dBSA) possess good stability in the intracellular environment, without the detachment of R6G from the nanoconjugates. These observations demonstrate the capability of HAuNFs@R6G@dBSA as dark-field and fluorescent imaging nanoprobes, which would combine the advantages of excellent photo-stability (i.e., negligible photobleaching or photo-blinking) conferred by the scattering imaging based on novel metal nanoparticles [32], alongside the high spatial resolution and rich information at the subcellular level provided by (confocal) fluorescent imaging [33].

### 2.5. Evaluation of Intracellular SERS Performance of HAuNFs-Containing Nanoconjugates

**Figure 5:**
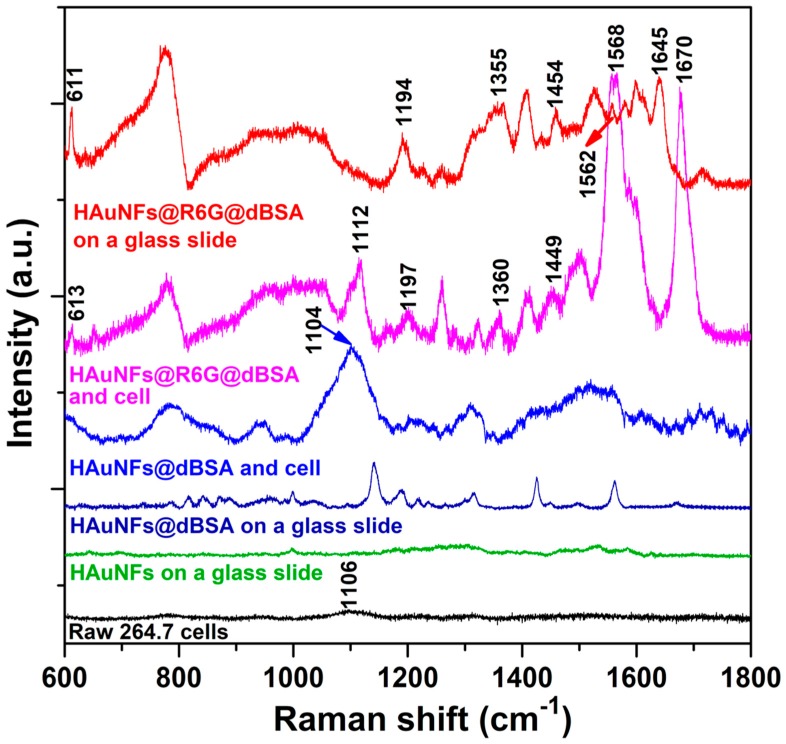
Raman spectra of HAuNFs, HAuNFs@dBSA, or HAuNFs@R6G@dBSA on a glass slide and individual Raw 264.7 cells treated with medium containing HAuNFs@dBSA, HAuNFs@R6G@dBSA, or medium only as a blank control. The spectra were recorded under the same measurement conditions, and have been shifted vertically for clarity in comparison. The spectra of nanostructures on a glass slide were collected by drop-casting 20 μL of the sample solution on glass slides and performing the measurements on the concentrated ring area post natural drying.

Beyond these two imaging modalities, intracellular SERS can provide additional information at the molecular level, thus, we have also evaluated the single-cell intracellular SERS performance of HAuNFs-containing nanoconjugates. The as-prepared HAuNFs showed few peaks in the Raman spectrum (Figure 5), because of the surfactant-free preparation and the degradation of the MO-FeCl_3_ nanofiber template. Raman peaks are distinguishable in the spectrum of HAuNFs@dBSA, implying the SERS effect of HAuNFs on the coated dBSA layer. HAuNFs@R6G@dBSA on a glass slide exhibited the main characteristic peaks of R6G, including 611 cm^−1^ (R6G: In-plane xanthene ring deformations), 1194 cm^−1^ (R6G: In-plane C-H bend), 1355 cm^−1^ (R6G: Xanthene ring stretch and in-plane C-H bend), 1454 cm^−1^ (R6G: Aromatic ring C-C stretch), 1562 cm^−1^ (R6G: Xanthene ring stretch and in-plane N-H bend), and 1645 cm^−1^ (xanthene ring stretch and in-plane C-H bend) [34,35,36]. When the spectrum was collected from a single cell, the cells incubated with medium only showed almost an absence of Raman signals, except a weak peak centered at 1106 cm^−1^ (DNA backbone) [37]. This peak was largely enhanced in the cells treated with HAuNFs@dBSA nanoconjugates, suggesting the intracellular SERS effect of HAuNFs-containing nanoconjugates, which enables sensitive detection of the chemical components in the intracellular microenvironment. The cells treated with the SERS tag (HAuNFs@R6G@dBSA) not only exhibited the peaks of cell biomolecules, but also the characteristic peaks of R6G, similar to the peaks observed in the Raman spectrum of these tags on a glass slide. The wide peaks from 820 to 1050 cm^−1^ in the spectra of HAuNFs@R6G@dBSA on a glass slide or with cells arise from the fluorescence of R6G, revealing that HAuNFs@R6G@dBSA in the cells retain the fluorescent features, which could be applied for intracellular fluorescent imaging, thus, corroborating the observations in Figure 4. The peaks at 1568 and 1670 cm^−1^ in the spectrum of the cell treated with SERS nanotags displayed a remarkable enhancement when compared with corresponding peaks at 1562 and 1645 cm^−1^ of the SERS nanotags on a glass slide. This spectral change is attributable to the variation in the fingerprints of R6G and dBSA caused by the local intracellular environment. The successful detection of SERS signals of R6G reporter molecules from a single cell suggests the capability of our HAuNFs@R6G@dBSA for uses as intracellular SERS nanoprobes.

## 3. Materials and Methods

### 3.1. Materials

Gold(III) chloride trihydrate (520918), Bovine Serum Albumins (BSA, A2058), and Rhodamine 6G (R6G, R4127) were purchased from Sigma-Aldrich (Haverhill, UK). Iron(III) chloride, hexahydrate (FeCl_3_·6H_2_O, A16231), l-(+)-Ascorbic acid (A15613), and methyl orange (17874) were purchased from Alfa Aesar (Heysham, UK)). Sodium borohydride (NaBH_4_, 10599010), nitric acid (70%, UN2031), and hydrochloric acid (37%, UN1789) were purchased from Fisher Scientific (Loughborough, UK). All chemicals were used without further purification. Milli-Q water (18.2 MΩ) was used in all experiments.

### 3.2. Characterisation

The UV-vis spectra were recorded with a Perkin-Elmer Model Lambda 35 spectrophotometer (Waltham, MA, USA). The fluorescent spectra were collected using an Edinburgh Instruments FLS 980 spectrometer (Livingston, UK). Transmission electron microscope (TEM) images were taken using TEM, Tecnai™ G^2^ Spirit TWIN/BioTWIN (Hillsboro, OR, USA), with an acceleration voltage of 120 kv. An atomic absorption spectrometer (Varian 240 fs, Palo Alto, CA, USA) was used to measure the concentration of gold nanostructures in dispersion.

### 3.3. Synthesis of HAuNFs

Vials were treated with aqua regia (nitric acid and hydrochloric acid in a molar ratio of 1:3) thoroughly rinsed with DI water, and dried in an 80 °C oven. Once dry, the vials were cooled to room temperature. For preparing HAuNFs with an SPR around 650 nm, FeCl_3_·6H_2_O (13.5 mg) was dissolved in methyl orange aqueous solution (1 mL, 5 mM) to form the template dispersions. The template dispersion (120 μL) and ascorbic acid aqueous solution (2 mL, 125 mM) were added sequentially into the HAuCl_4_ aqueous solution (2 mL, 5 mM). Following an undisturbed growth for 45 min at room temperature, the reaction dispersion was centrifuged (188 *g* × 10 min). After the removal of the supernatant, the pellet was washed with Milli-Q water twice. The final product was collected in Milli-Q water.

### 3.4. Surface Modification

Conjugation of HAuNFs and R6G: HAuNFs (O.D. ~1) was added into the aqueous solution of R6G (50 μM) at a 1:1 volume ratio and allowed to stay overnight. The products were collected by centrifugation (188 *g* × 10 min) and re-dispersed in Milli-Q water. The redispersion-centrifugation cycle was performed 3 times to remove free R6G. The resultant pellet was redispersed in Milli-Q for UV-vis spectrum measurement.

Coating with denatured BSA (dBSA): HAuNFs@R6G nanoconjugates (O.D. ~1) or HAuNFs (O.D. ~1) was dispersed in the as-prepared dBSA solution at a 1:1 volume ratio. dBSA was prepared via the reaction of BSA and NaBH_4_ to convert disulfide bonds in BSA into thiol groups, following a reported method [38]: 2 mg of NaBH_4_ was added into the aqueous solution of BSA (10 mL, 3.3 mg/mL) with vigorous magnetic stirring. The reaction was kept at room temperature for 2 h and then 70 °C until no gas (H_2_) was observed.

### 3.5. Cell Culture

RAW 264.7 cells (ATCC-TIB-71, purchased from Invitrogen Life Technologies (Carlsbad, CA, USA) were cultured in Roswell Park Memorial Institute (RPMI) medium (61870-010, purchased from Life Technologies), supplied with Fetal Bovine Serum 10%. The cells were incubated at 37 °C in a humidified environment supplied with 5% CO_2_.

### 3.6. Cytotoxicity Assay

The cell viability assays (Cell Counting Kit-8, CCK-8) were conducted following the instructions of the manufacturers. Cells were first seeded in 96-well plates (3599, purchased form Corning (Corning, NY, USA) at a density of 5000 cells per well. After 24 h of incubation in 100 μL of RPMI 1640 medium at 37 °C, 10 μL of RPMI 1640 medium containing different dosages of HAuNFs was introduced into each well (the dosage in the Results and Discussion part means the final concentration of HAuNFs contained in each well). Following the treatment of 24 h, CCK-8 agent (10 μL) was introduced in each well. After another incubation for 4 h, the absorbance was measured on a micro-plate reader (Mithras LB 940) at the wavelength of 450 nm. The cell viability was expressed as the percentage of optical density (OD) as compared with that of the blank control. A blank RPMI 1640 medium containing HAuNFs at a corresponding dose was used as the background, the OD of which was subtracted for getting rid of the optical interference from HAuNFs.

### 3.7. Optical Microscopy Imaging

Raw 264.7 cells were seeded onto 20 mm glass coverslips in a 6-well plate (Corning, 3516) at a certain density of 1 × 10^5^ cells/well and allowed to grow for 48 h. The medium was then replaced with 2 mL of a medium containing HAuNFs or HAuNFs-containing nanoconjugates at the concentration of 50 μg Au/mL. After 12 h of incubation and the removal of the culture medium, the cells on the coverslip were washed with DPBS (14190-094, purchased from Life Technologies) twice, fixed with DPBS containing 4% paraformaldehyde, and then repeatedly washed with DPBS for several times. The fixed coverslips were mounted using Mowiol^®^ 4-88 (81,381 Aldrich) and sealed onto glass slides. Optical microscopy imaging (bright field, dark field, and fluorescence) was performed with an inverted microscope (Nikon Eclipse Ti-E, Nikon UK Limited, Surrey, UK) and an oil coupled 100× objective (CFI Plan Fluor, Nikon UK Limited, Surrey, UK). Nile red channel (excitation 559 ± 34 nm, emission 630 ± 69 nm) was used for fluorescence imaging. Images were recorded using a 5 Megapixel colour camera (DS-Fi1, Nikon UK Limited, Surrey, UK) and saved using NIS-Elements Documentation (Nikon UK Limited, Surrey, UK) software.

### 3.8. Transmission Electron Microscopy (TEM) Imaging of Cell Sample

Raw 264.7 cells were seeded in a 6-well plate (Corning, 3516) at a certain density of 1 × 10^5^ cells/well and allowed to grow for 48 h, followed by treatment with HAuNFs (50 μg/mL) in media for 12 h at 37 °C. After being washed with PBS, the cells were detached and centrifuged. The fixation of cells was performed by treating the cell pellet with glutaraldehyde (2.5%) in phosphate buffer (0.1 M) for 2.5 h. The fixed cells were dehydrated with an alcohol series (20%, 40%, 60%, 80%, and 100% twice) for 20 min in each cycle and then embedded in Araldite resin overnight at 65 °C. A Section of 70 nm was deposited on TEM grid for TEM imaging.

### 3.9. Intracellular SERS

Raw 264.7 cells were seeded onto 20 mm glass coverslips in a 6-well plate (Corning, 3516) at a certain density of 1 × 10^5^ cells/well and allowed to grow for 48 h. The medium was then replaced with 2 mL of a medium containing HAuNFs or HAuNFs-containing nanoconjugates at the concentration of 50 μg Au/mL. After incubation for 12 h and the removal of the culture medium, the cells on the coverslip was washed with DPBS twice, fixed with DPBS containing 4% paraformaldehyde, and then repeatedly washed with DPBS. The fixed coverslips were sealed onto glass slides, without being mounted, to exclude the possible interference of a Raman signal from the mounting glue (Mowiol^®^ 4-88). Raman spectra were taken on a Horiba-Jobin-Yvon LabRam HR, with 633 nm excitation. All measurements were conducted using a 100× objective, and a laser power of 0.18 mW (on the sample) with an integration time of 30 s.

## 4. Conclusions

In this work, we have carefully tuned the optical properties of our HAuNFs to construct HAuNFs@R6G@dBSA nanoconjugates, with the SPR absorption matching the 633 nm excitation wavelength for optimized SERS effect. The cytotoxicity test using the CCK-8 cell viability assay reveals the good bio-compatibility of the as-prepared HAuNFs on Raw 264.7 cells. Both dark-field and TEM images showed the effective cellular uptake of HAuNFs by Raw 264.7 cells. Subsequently, we coated HAuNFs with R6G and dBSA. The obtained nanoconjugates (HAuNFs@R6G@dBSA) have been demonstrated to be applicable as a trimodal intracellular nanoprobe combining dark-field, fluorescent imaging, and SERS functions.

## Supplementary Materials

Supplementary materials can be found at http://www.mdpi.com/1422-0067/19/8/2327/s1.
