# Supplementary material for: Developing Hollow-Channel Gold Nanoflowers as Trimodal Intracellular Nanoprobes
Source: Int J Mol Sci. 2018 Aug 8;19(8):2327. doi: 10.3390/ijms19082327 (PMC6121537; doi:10.3390/ijms19082327)
Supplement: Supplementary file 1 [file ijms-19-02327-s001.zip › ijms-326092-supplementary.pdf]

Supplementary Materials: Information: Developing Hollow-channel Gold Nanoflowers as Trimodal Intracellular Nanoprobes

Sunjie Ye, May C. Wheeler, James R. McLaughlan, Abiral Tamang, Christine P. Diggle, Oscar Cespedes, Alex F. Markham, Patricia Louise Coletta and Stephen D. Evans

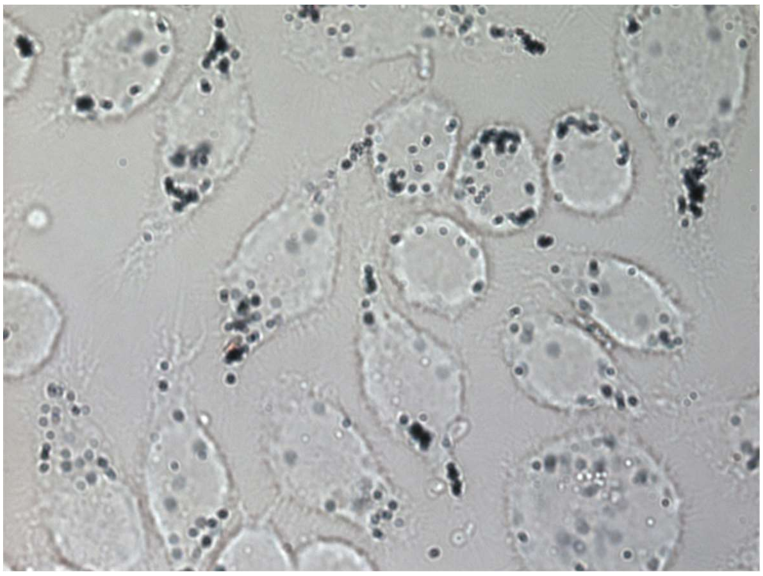

Figure S1. Bright field microscopy image of Raw 264.7 cells treated with HAuNFs@R6@dBSA.

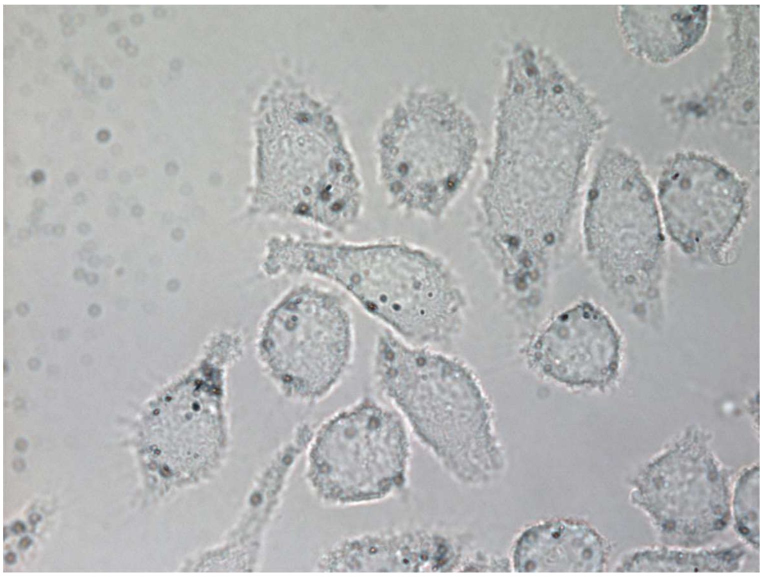

Figure S2. Bright field microscopy image of Raw 264.7 cells treated with HAuNFs@dBSA.
